# Supplementary material for: Impact of deltamethrin-resistance in Aedes albopictus on its fitness cost and vector competence
Source: PLoS Negl Trop Dis. 2021 Apr 27;15(4):e0009391. doi: 10.1371/journal.pntd.0009391 (PMC8104426; doi:10.1371/journal.pntd.0009391)
Supplement: Attachment — Submitted filename: Review PNTD.docx [file pntd.0009391.s001.docx]

Insecticide resistance management (RM) strategies such as rotation of insecticide classes rely on resistance having a fitness cost causing selection against resistance alleles in the absence of insecticide selection pressure. Therefore, understanding the fitness cost that resistance imposes on the mosquito population is a key prerequisite to effective IRM as it could contribute to implement suitable control measures.

In this study, The authors firstly established a laboratory strain of Aedes albopictus resistant to deltamethrin, and compared the fitness cost and vector competence of DENV-2 between the resistant strain and the susceptible strain

The results obtained may provide a basis for the rational use of insecticides to delay the development of resistance by rotation or discontinuation. Such information is important for resistance management strategies. I have only minor comments which need to be addressed before the article could be suitable for publication.

**Minor comment :**

Line29-30 : is the difference significant ?

Line 31&32&38 : add the p-value

Line 57-59 : to be reformulated

Line 280-282 : ‘’ deltamethrin 281 resistance may affect the development of Aedes albopictus by prolong the time for larvae to develop into adult mosquitoes’’. the authors did not found any difference meaning that this resistance has no effect on the development therefore, the conclusion is wrong.

Line 398 replace ‘’ To assessed’’ by ‘’to assess’’
