# Supplementary material for: Impact of deltamethrin-resistance in Aedes albopictus on its fitness cost and vector competence
Source: PLoS Negl Trop Dis. 2021 Apr 27;15(4):e0009391. doi: 10.1371/journal.pntd.0009391 (PMC8104426; doi:10.1371/journal.pntd.0009391)
Supplement: Attachment — Submitted filename: PNTD-R1 reply letter.docx [file pntd.0009391.s002.docx]

Dear Editor and Reviewers,

Thank you for your letter and for the reviewers’ comments concerning our manuscript entitled “Impact of deltamethrin-resistance in *Aedes albopictus* on its fitness cost and vector competence ”(PNTD-D-20-02221). Those comments are all valuable and very helpful for revising and improving our manuscript, as important guiding significance to our researches. We have studied comments carefully and have made correction which we hope meet with approval. Revised portion are marked in red in the manuscript.

The main corrections in the paper and the responds to the reviewers’ comments are as following:

**To Reviewer #1**

**Minor Comment 1:** Line29-30 : is the difference significant ?

**Response:** Thanks for your question. We have added P values in the abstract section to better present our results (line 28-29). In addition, we are sorry that we calculated the average development time of pupa incorrectly before, and this has been corrected. The average development time of Lab-R and Lab-S in larvae was 9.7 days and 8.2 days (*P* < 0.005), and in pupae was 2.0 days and 1.8 days respectively (*P* > 0.05), indicating that deltamethrin resistance prolongs the larval development time rather than pupal development time of resistant mosquitoes.

**Minor Comment 2:** Line 31&32&38 : add the p-value

**Response:** Thanks for your suggestion .The requested change was made (line 39-40).

**Minor Comment 3:** Line 57-59 : to be reformulated

**Response:** Thank you for your advice. We have revised the manuscript to better summarize our views (line 59-61).

**Minor Comment 4:** Line 280-282 : “deltamethrin resistance may affect the development of *Aedes albopictus* by prolong the time for larvae to develop into adult mosquitoes’’. the authors did not found any difference meaning that this resistance has no effect on the development therefore, the conclusion is wrong.

**Response:** Thanks for your comment. The result here is ambiguity in our description. The results of line 274-277 showed that the larval development time was prolonged. We also made a supplement in the answer to the first question based on your question. We have changed the conclusion in line 284-286. Deltamethrin resistance may affect the development of *Aedes albopictus* by prolong the time form larvae to develop into pupae.

**Minor Comment 5:** Line 398 replace “To assessed’’ by ‘’to assess’’

**Response:** Thank you for your advice. We have changed it in the revised manuscript.

**To Reviewer #2**

**Comment 1:** The conclusions are correct and carefully drawn and the fact that the two strains have similar genetic background is in support. However, the (negative in this case) correlation between insecticide resistance and fitness cost/infectivity may not be a general correlation between phenotypes, but specific for the mechanism selected by the approach taken (extensive laboratory selection for many generation), in that case the conclusion might also be more generally applicable and valid.
It would be very useful, with not much more effort I believe, if the authors sequence the pyrethroid target in the regions *kdr* type mutations have been reported in this species in the two strains, to find out if target site resistance or no target resistance is responsible for the phenotype, and thus open the applicability of their conclusion. If resistance has been characterized already in other study, then please just cite the study and mention the dominant mechanism.

**Response:** We appreciate your suggestion. In this study, we focused on the impact of deltamethrin resistance on vectorc competence and fitness cost of *Aedes albopictus*. The *kdr* detection and correlation analysis you proposed are ongoing work that we are also interested in, and these data will be presented in another paper.

**Comment 2:** It would be useful to please sequence the pyrethroid target in the region of *kdr* type mutations previously found in *Ae albopictus*, to define if target site resistance is responsible or not for the phenotype, and thus open the applicability of the correlation conclusions.

**Response:** Thank you for your advice. Like the previous answer, further analysis and research is under way.

**To Reviewer #3**

**Comment 1:**

1) Table1，add CM (%)(95% CI).

2) Line273, Which generation started to make a difference？the value of t OR F？What caused the difference? Is there a correlation Developing time and level of resistance？
3) Line280 The value of t OR F？
4) Table 2 S1 why DENV-2 copies in serum were observed in DAY1 and DAY5, but negative in DAY3 and DAY7, Is this a detection problem or what? The same problem appeared in S4.

**Response:** Thank you very much for your questions.

1) The requested change in Table 1 was made, and sorry that we miswrote the LC_50_ of Lab-S strain and have corrected it.

2) It is an interesting question about the correlation between developing time and level of resistance. But we only studied the vector competence of the 27th generation of resistant strain and analyzed its fitness cost. We did not observe the development time of other generations of resistant mosquitoes. We were also interested in the reasons for the differences in developmental time. As seen in line 446, we hypothesized that it might be related to the resistance mechanism of mosquitoes: the alteration of target site and the alteration of metabolic enzyme activity. Further study need to be conducted.

3) The value of t or F have been added.

4) The amount of blood we take from suckling mice is relatively few but all were quantitative. We think it could be a detection problem at a low level of infection in the mice. Therefore, we detected multiple samples and multiple times to rule out false positives/negatives in the test. Finally, due to the deficiency of blood detection results, we supplemented the brain tissue data to improve the persuasion of this part of our experiment (horizontal transmission).

**Comment 2:**

1) Line35.36 Is there no difference between resistant and sensitive strains during the early period of infection? Is it generally believed that there is a difference? why?
2) The vertical and horizontal transmission of resistant strains is lower than that of susceptible strains. Why is the level of drug resistance increasing worldwide, but dengue outbreaks are more frequent?

**Response:** Thank you for your questions.

1) In our study, there was no statistical difference in the susceptibility of the two strains during early period of dengue infection. The interaction between different vectors and different viruses will be different, and the conclusions of the studies have been reported were different. As was mentioned in our discussion (line 463), the vector competence of resistant *Culex quinquefasciatus* for West Nile virus was increased while for Rift Valley Fever virus did not change, suggesting different interactions between different pathogens and vectors. (Atyame CM, et al.Proc Biol Sci. 2019 Jan 16;286(1894):20182273.)

2) The prevalence of dengue fever is affected by many factors, including climate change, population movement, the feeding habits of mosquitoes and so on. Mosquitoes can survive under selecting pressure of insecticides by developing resistance in order to restore vector population density, making the rapid development of insecticides resistance have become a problem for vector control. In the present study, although the vector competence of resistant *Aedes albopictus* to DENV-2 is reduced, it can still complete the external incubation period and transmit dengue virus. We can infer that deltamethrin-resistant *Aedes albopictus* remains a potential vector for dengue virus transmission.

**Once again, we thank you very much for your comments and suggestions, and hope the corrections will meet with approval..**

Best regards

Xiao-Guang Chen
